# Contemporary posterior surgical approach in total hip replacement: still more reoperations due to dislocation compared with direct lateral approach? An observational study of the Swedish Hip Arthroplasty Register including 156,979 hips

**DOI:** 10.1080/17453674.2019.1610269

**Published:** 2019-05-07

**Authors:** Oscar Skoogh, Georgios Tsikandylakis, Maziar Mohaddes, Szilard Nemes, Daniel Odin, Peter Grant, Ola Rolfson

**Affiliations:** a Department of Orthopaedics, Sahlgrenska University Hospital;; b Swedish Hip Arthroplasty Register;; c Department of Orthopaedics, Institute of Clinical Sciences, Sahlgrenska Academy, University of Gothenburg, Sweden

## Abstract

**Background and purpose —** The direct lateral approach (DLA) and the posterior approach (PA) are the most common surgical approaches in total hip replacement (THR) in Sweden. We investigated how the relationship between surgical approach and risk of reoperation due to dislocation has evolved over time.

**Patients and methods** — Data were extracted from the Swedish Hip Arthroplasty Register from 1999 to 2014. We selected all THRs due to osteoarthritis with head sizes 28, 32, and 36 mm that were performed with either the DLA or the PA. Resurfacing prostheses were excluded. Kaplan–Meier curves for risk of reoperation due to dislocation and all-cause for the 2 surgical approaches were compared for 2 periods (1999–2006 and 2007–2014) up to 2 years postoperatively. We used Cox regression for sex, age, type of fixation, and head size to determine hazard ratios (HR) with DLA set as reference.

**Results —** 156,979 THRs met the selection criteria. In 1999–2006, the PA was associated with increased risk of reoperation due to dislocation (HR 2.3, 95% CI 1.7–3.0) but there was no difference in the risk of all-cause reoperation (HR 1.1, CI 0.9–1.2). In 2007–2014 there was no statistically significant difference in the risk of reoperation due to dislocation (HR 1.2, CI 0.9–1.6) but the risk of all-cause reoperation was lower (HR 0.8, CI 0.7–0.9) for the PA.

**Interpretation —** This study confirms historic reports on the increased risk of early reoperations due to dislocations using the PA compared with the DLA. However, in contemporary practice, the higher risk of reoperation due to dislocation associated with PA has declined, now being similar to that after DLA. We believe improved surgical technique for the PA may explain the results. Surprisingly, the PA was associated with lower risk of all-cause reoperation in 2007–2014. This finding warrants further investigation.

The posterior approach (PA) and the direct lateral approach (DLA) are the 2 principal surgical approaches for total hip replacement (THR) in Sweden (Kärrholm et al. 2017). There is no consensus regarding the optimum surgical approach to be used in primary THR (Jolles and Bogoch 2006). A majority of the dislocations occurs within the first 2 years postoperatively and is 1 of the most common early complications following THR (Hailer et al. 2012, Gausden et al. 2018). Multiple risk factors for dislocation have been identified such as surgical approach, orientation of components, femoral head size, previous surgery, age, sex, BMI, indication for surgery, and comorbidities such as neurological disability and spinal disease (Bystrom et al. 2003, Berry et al. 2005, Patel et al. 2007, Hailer et al. 2012, Fessy et al. 2017, Seagrave et al. 2017, Gausden et al. 2018).

The PA has been associated with higher dislocation rate in comparison with the DLA (Robinson et al. 1980, Woo and Morrey 1982, Demos et al. 2001, Masonis and Bourne 2002, Bystrom et al. 2003, Jolles and Bogoch 2006, Hailer et al. 2012, Lindgren et al. 2012). However, enhanced soft tissue repair and improved surgical technique for the PA lower dislocation rates (Pellicci et al. 1998, White et al. 2001, Soong et al. 2004, Suh et al. 2004, Kwon et al. 2006, Kim et al. 2008, Zhou et al. 2017).

According to the Swedish Hip Arthroplasty Register (SHAR), the PA and the DLA are used in 95% to 99% of the primary THRs in Sweden. The DLA that has increased from 37% in 1999 to 48% in 2014 at the expense of the PA, which has decreased from 60% in 1999 to 51% in 2014 (Figure 1).

**Figure 1.**
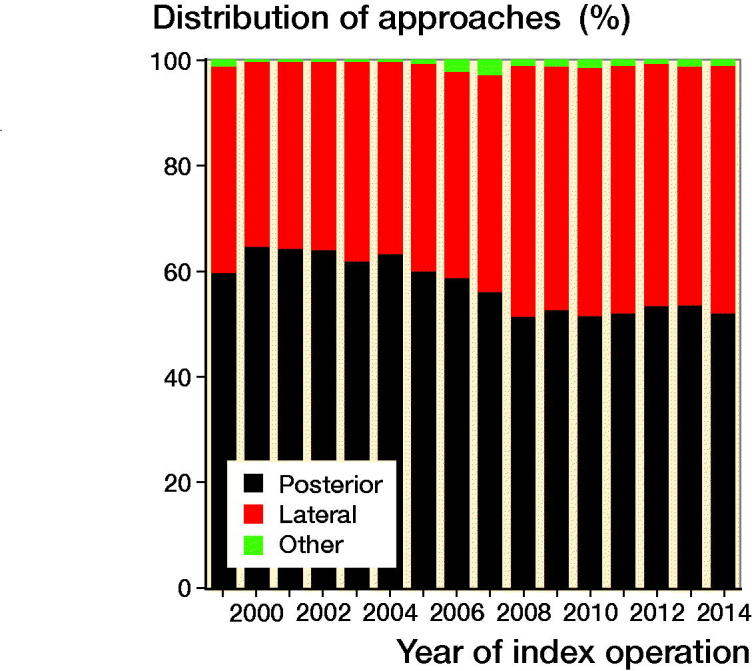
Distribution of posterior, direct lateral and other approaches among THRs performed in Sweden from 1999 to 2014.

To our knowledge there are no studies comparing trends for the risk of reoperation due to dislocation for the PA and the DLA over a long period of time. In the light of improvements in surgical technique, we investigated how the relationship between surgical approach and risk of reoperation due to dislocation has evolved over time.

## Patients and methods

**Figure 2.**
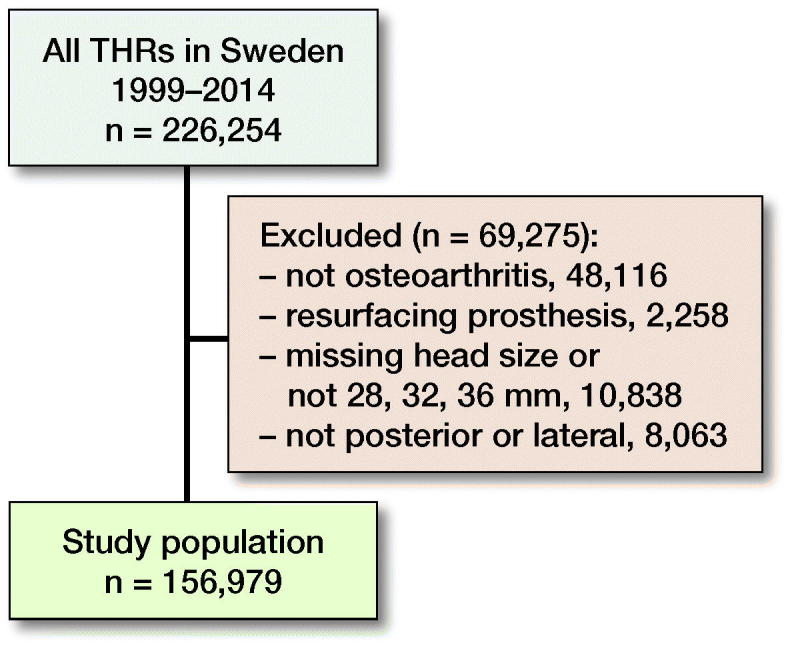
Patient selection flowchart. In order to reduce heterogeneity, the study population was defined according to preset selection criteria. Starting with all THRs in Sweden between 1999–2014 we applied the selection criteria to step-wise filter out relevant surgeries.

Since 1979, the SHAR has collected data from all units in Sweden performing THR. The completeness of primary registrations to the SHAR is 98% to 99%, and 93% for revisions (Kärrholm et al. 2017). Data we extracted from the Register included all patients who had primary THR due to osteoarthritis between 1999 and 2014. Resurfacing prostheses and head sizes other than 28, 32, and 36 mm were excluded. Only patients operated with PA or DLA were included (Figure 2). 156,979 hips met the selection criteria. SHAR started collecting data on ASA class and BMI in 2008 (Table 1).

**Table 1.** Study demographics. Direct lateral and posterior approaches compared

	1999–2006			2007–2014		
	Lateral n = 22,507	Posterior n = 37,691	p-value	Lateral n = 44,933	Posterior n = 51,848	p-value
Age, mean (SD)	68.7 (9.9)	69.6 (9.5)	< 0.001 **^a^**	68.8 (9.7)	69.2 (9.5)	< 0.001 **^a^**
Female sex, n (%)	12,829 (57)	21,297 (57)	0.2 **^b^**	26,329 (59)	29,401 (57)	< 0.001 **^b^**
ASA-class, n (%)						< 0.001 **^b^**
I				9,994 (22)	10,160 (20)	
II				23,902 (53)	26,474 (51)	
III				5,599 (13)	7,069 (14)	
IV				143 (0.3)	168 (0.3)	
V				0 (0)	3 (0.0)	
Missing				5,295 (12)	7,974 (15)	
BMI, mean (SD)				27.3 (5.1)	27.5 (5.2)	< 0.001 **^a^**
Missing, n (%)				6,236 (14)	8,451 (16)	

**^a^**ANOVA, **^b^**Chi-squared test

### Outcome measures

Primary outcome measure was first reoperation due to dislocation as reported to the register within 2 years following index surgery. Secondary outcome was all-cause reoperation within 2 years. A reoperation was defined as any further open surgery to the hip, regardless of implant components being removed, exchanged, added, or not. Thus, surgeries such as gluteus maximus repair, tenotomy, and hip arthroscopy were included among reoperations.

### Statistics

We used 1-way ANOVA test (with equal variance assumption) for continuous demographic variables (age and BMI) and chi-square test (with continuity correction) for categorical variables (sex, fixation, head size, ASA, and cause of reoperation). Kaplan–Meier curves for posterior and direct lateral surgical approaches were compared for 2 different time periods (1999–2006 and 2007–2014) until 2 years follow-up. We used data from both operations if patients were bilaterally operated during the study period; the violation of the assumption of independent observations was considered not to have any practical implications (Ranstam et al. 2011). Each hip was followed from primary THR to first reoperation. Hips were censored at death, reoperation, or at 2 years after primary surgery, whichever came first. For the dislocation analyses, all other first reoperations were censored. Cox regression analyses were used to compare hazard ratios (HR) with and without adjustments with 95% confidence intervals (CI). We adjusted for sex, age, fixation method (cemented, uncemented, hybrid, and reversed hybrid) and head size (except for 1999–2006 as 99% of hips had 28-mm head size). Proportional assumption was checked graphically.

Divided by year of surgery and for PA and DLA separately, we calculated Kaplan–Meier survival estimates with reoperation due to dislocation at 2 years as endpoint. The uncertainty of the Kaplan–Meier estimates was indicated by CIs. Linear regression was used to determine whether the linear trend was statistically significant. A p-value of less than 0.05 indicated statistical significance.

R software version 3.4.2 (R Core Team 2017) was used for all analyses with “survival” packages “A Package for Survival Analysis in S” and “ggplot2” (Therneau 2015, Wickham 2016).

### Ethics, funding, and potential conflicts of interest

The Regional Ethical Review Board in Gothenburg approved the study (entry number 804-17). The study received grants from the Handlaren Hjalmar Svensson fund. Grants from the Swedish state under the agreement between the Swedish government and the county councils, the ALF-agreement (ALFGBG–522591) also contributed to the study. The authors have no conflicts of interest.

## Results

**Figure 3.**
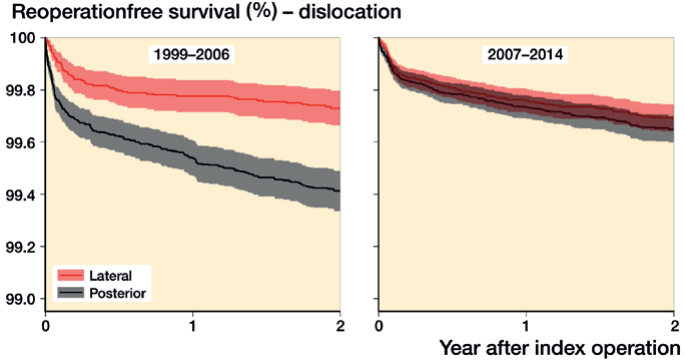
Kaplan Meier estimates for not being reoperated due to dislocation within 2 years for posterior and direct lateral surgical approaches during 1999–2006 and 2007–2014. Shaded area are 95% confidence intervals.

As indicated in the Kaplan–Meier curves, the risk of reoperation due to dislocation in 1999–2006 was statistically significantly higher for the PA almost directly postoperatively (Figure 3). PA was associated with higher risk of reoperation due to dislocation in 1999–2006 (HR 2.3, CI 1.7–3.0) but not in 2007–2014 (HR 1.2, CI 0.9–1.6) compared with the DLA (Table 2).

**Table 2.** Cox regression analyses were used to compare hazard ratio (HR) for reoperation due to dislocation and reoperation due to all causes within 2 years with and without adjustments for time periods 1999–2006 and 2007–2014

	1999–2006		2007–2014	
Reoperation cause	HR (95% CI)	p-value	HR (95% CI)	p-value
**Dislocation**				
Unadjusted	2.2 (1.7–2.9)	< 0.001	1.1 (0.9–1.5)	0.3
Adjusted				
Posterior approach (ref. direct lateral approach)	2.3 (1.7–3.0)	< 0.001	1.2 (0.9–1.6)	0.2
Age	1.0 (1.0–1.0)	< 0.001	1.0 (1.0–1.0)	0.004
Female (ref. male)	0.8 (0.6–1.0)	0.03	0.9 (0.7–1.1)	0.4
Hybrid (ref. cemented)	1.1 (0.5–2.4)	0.8	2.0 (0.9–4.6)	0.1
Reverse hybrid (ref. cemented)	1.6 (0.8–3.1)	0.2	0.9 (0.6–1.5)	0.8
Uncemented (ref. cemented)	1.8 (1.0–3.3)	0.07	2.7 (1.9–3.9)	< 0.001
32-mm head (ref. 28-mm head)	N/A		0.7 (0.5–0.9)	0.01
36-mm head (ref. 28-mm head)	N/A		0.6 (0.4–1.1)	0.09
**All causes**				
Unadjusted	1.0 (0.9–1.2)	0.8	0.8 (0.7–0.9)	< 0.001
Adjusted				
Posterior approach (ref. direct lateral approach)	1.1 (0.9–1.2)	0.4	0.8 (0.7–0.9)	< 0.001
Age	1.0 (1.0–1.0)	0.002	1.0 (1.0–1.0)	< 0.001
Female (ref. male)	0.8 (0.7–0.9)	< 0.001	0.7 (0.7–0.8)	< 0.001
Hybrid (ref. cemented)	1.1 (0.8–1.6)	0.6	1.0 (0.7–1.6)	1.0
Reverse hybrid (ref. cemented)	1.9 (1.4–2.6)	< 0.001	1.5 (1.3–1.7)	< 0.001
Uncemented (ref. cemented)	1.5 (1.1–2.0)	0.02	1.9 (1.6–2.2)	< 0.001
32-mm head (ref. 28-mm head)	N/A		1.0 (0.9–1.1)	0.9
36-mm head (ref. 28-mm head)	N/A		1.1 (0.9–1.4)	0.5

**Figure 4.**
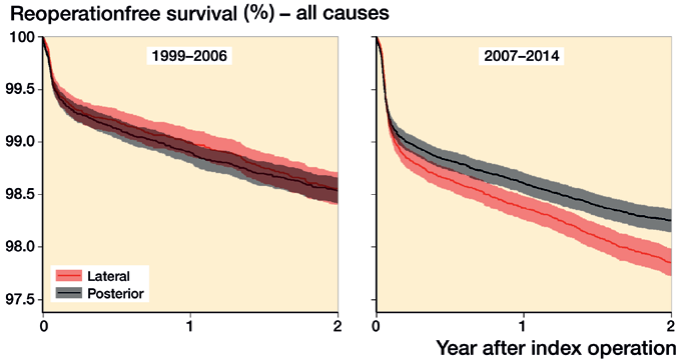
Kaplan-Meier estimates for not being reoperated up to 2 years (all causes) for posterior and direct lateral surgical approaches during 1999–2006 and 2007–2014. Shaded area are 95% confidence intervals.

All-cause reoperation Kaplan–Meier estimates for the 2 approaches were similar for the first period. For the period 2007–2014, however, the Kaplan–Meier curves indicated a higher risk of reoperation due to all causes for the DLA starting at approximately 1 year postoperatively (Figure 4).

PA was associated with similar risk of all-cause reoperation in 1999–2006 (HR 1.1, CI 0.9–1.2) compared with the DLA (Table 2). In 2007–2014, PA was associated with statistically significantly lower risk of reoperation due to all causes (HR 0.8, CI 0.7–0.9) compared with the DLA (Table 2).

**Figure 5.**
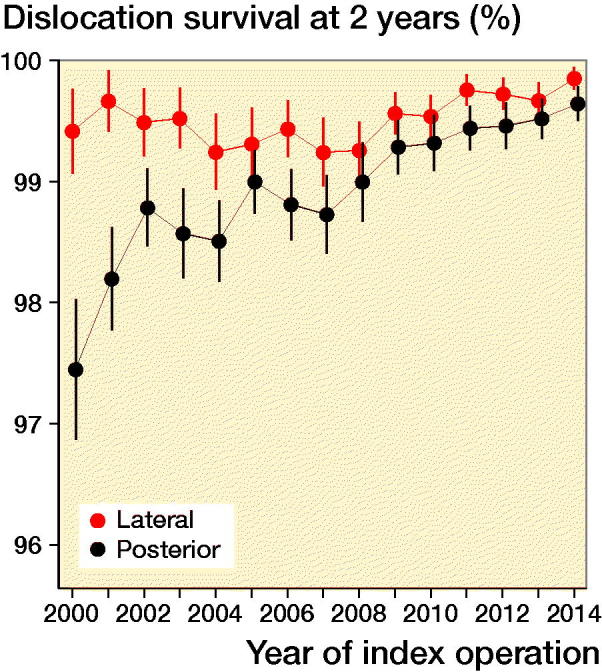
Annual Kaplan Meier estimates (and 95% confidence intervals) for not being reoperated due to dislocation at 2 years after primary THR for posterior and direct lateral approach. Linear regression was used to investigate if the linear trend was statistically significant. Lateral, p < 0.05 and posterior, p < 0.001.

Split by year for primary surgery, the trend analysis of Kaplan–Meier estimates for not being reoperated due to dislocation at 2 years demonstrated positive linear trends for both the DLA (p < 0.05) and the PA (p < 0.01) (Figure 5).

## Discussion

This study confirms historic reports on the increased risk of early reoperations due to dislocations using the PA compared with the DLA in primary THR due to OA. However, in contemporary practice, the higher risk associated with PA had declined and did not entail a statistically significant increased risk of reoperation due to dislocation within 2 years from primary surgery compared with DLA. Surprisingly, the PA was associated with lower risk of reoperation due to all causes. Despite differences in head size, fixation type, and demography between groups, adjusting for confounders did not alter the results.

In this nationwide observational study, the rate of reoperations due to dislocation within 2 years following THR for OA was 0.6% in 1999–2006 and 0.4% in 2007–2014 for the PA, and 0.3% in 1999–2006 and 0.3% in 2007–2014 for the DLA. In the meta-analyses by Kwon et al. (2006) regarding 11 papers between 1997 and 2004 the dislocation rate for the DLA was 0.4% while it was 1.0% for the PA. In the review by Masonis and Bourne (2002) of 14 studies between 1976 and 2001 involving 13,203 primary THRs the dislocation rate was estimated as 6 times higher for the PA (3%) than the DLA (0.5%). According to a study by Hailer et al. (2012) with data from the SHAR on 78,098 THRs in 61,743 patients performed between 2005 and 2010 there was a 1.3 times increased relative risk of revision due to dislocation for the PA compared with the DLA with mean follow-up of 2.7 (0–6) years. However, in our study PA was not associated with higher risk of reoperation due to dislocation in 2007–2014.

There are important differences between ours and other studies when it comes to, e.g., selection criteria, time period, follow-up time, and use of components that have to be considered when comparing. We believe there has been an ongoing refinement of the surgical technique in THR over the years. For instance, in our study we found an extensive increase in head sizes larger than 28 mm in 2007–2014 compared with 1999–2006 (Table 3). According to a study by Berry et al. (2005) including 21,047 primary THRs performed in a single institution between 1969 and 1999, the relative risk of dislocation was 1.3 for 28 mm heads compared with 32 mm heads. This is consistent with our study where the 32 mm heads were associated with a statistically significantly lower risk of reoperation due to dislocation up to 2 years (HR 0.7, CI 0.5–0.9) compared with 28 mm heads (Table 2). However, 36 mm heads were not associated with lower risk of reoperation due to dislocation (HR 0.6, CI 0.4–1.1) in our study (Table 2). Larger head size and implant use may certainly explain some of the overall improvement in reoperation rates within 2 years for the 2 approaches investigated. However, we believe it is unlikely that the use of larger head size accounts for all the improvement. In 1999–2006, more than 99% of the THRs in Sweden were performed with 28 mm heads for both DLA and PA. Divided by year of primary surgery, improvement trend in annual Kaplan–Meier estimates was much more pronounced for PA compared with DLA (Figure 5). The increased reoperation-free survival for PA was evident already in 1999–2006. In those years, more than 99% of THRs were operated with 28 mm heads. Hence, the improvement for the PA in 1999–2006 is not attributable to use of larger head sizes. As discussed below, improved surgical technique may contribute to the positive trend for PA.

**Table 3.** Distribution of method of fixation, head size and different causes for reoperation within 2 years for direct lateral and posterior approaches during 1999–2006 and 2007–2014. Chi-squared test was used. Values are frequency (%)

	1999–2006			2007–2014		
	Lateral n = 22,507	Posterior n = 37,691	p-value	Lateral n = 44,933	Posterior n = 51,848	p-value
Method of fixation			< 0.001			< 0.001
Cemented	18,171 (81)	35,050 (93)		30,919 (69)	37,532 (72)	
Hybrid	1,219 (5.4)	869 (2.3)		609 (1.4)	933 (1.8)	
Reverse hybrid	1,002 (4.5)	908 (2.4)		5,975 (13)	6,272 (12)	
Uncemented	2,115 (9.4)	864 (2.3)		7,430 (17)	7,111 (14)	
Head size (mm)			< 0.001			< 0.001
28	22,386 (99)	37,479 (99)		22,239 (50)	19,799 (38)	
32	119 (0.5)	179 (0.5)		21,885 (49)	27,586 (53)	
36	2 (0.0)	33 (0.1)		809 (1.8)	4,463 (8.6)	
Reoperations within 2 years			< 0.001			< 0.001
Aseptic loosening	43 (0.2)	37 (0.1)		111 (0.2)	81 (0.2)	
Fracture	41 (0.2)	70 (0.2)		96 (0.2)	147 (0.3)	
Infection	124 (0.6)	180 (0.5)		486 (1.1)	441 (0.9)	
Dislocation	60 (0.3)	220 (0.6)		136 (0.3)	185 (0.4)	
Other	52 (0.2)	42 (0.1)		138 (0.3)	68 (0.1)	
Not reoperated	22,187 (99)	37,142 (99)		43,966 (98)	50,926 (98)	

Lindgren et al. (2014) used the SHAR to study 42,233 patients undergoing primary THR for OA operated between 2002 and 2010 and found that the PA was associated with slightly better patient-reported outcomes compared with the DLA. Hence, we believe the possible difference in PROMs should be considered in the choice of THR approach in OA patients.

To our knowledge there is no other study that has compared dislocation survival trends for the DLA and PA for such a long period of time. Our data from the SHAR showed statistically significant positive linear trends for both surgical approaches regarding the risk of being reoperated due to dislocation within 2 years after primary THR for OA in Sweden (Figure 5). This improvement over time was most apparent for the PA.

Since its inception in 1979, Swedish orthopedic surgeons have been influenced by the reports of SHAR. Register findings discussed at internal meetings in the mid-2000s indicated an increased risk of revision for the PA and, as demonstrated here, the use of the DLA increased at the expense of the PA. Between 2001 and 2006, a large body of research, including clinical trials, meta-analyses, and literature reviews, suggested that improved surgical technique with soft tissue repair following the PA in primary THR would reduce the risk of dislocation (Masonis and Bourne 2002, Mahoney and Pellicci 2003, Soong et al. 2004, Suh et al. 2004, Kwon et al. 2006). However, Hailer et al. (2012) concluded that patients with femoral neck fracture or osteonecrosis of the femoral head were at higher risk of dislocation and raised the question as to whether patients belonging to risk groups should be operated using lateral approaches. Furthermore, in a study by Enocson et al. (2009) on 713 consecutive hips, the use of the anterolateral approach for THR in patients with femoral neck fractures was advocated.

In the 2011 annual report, the SHAR reported specifically on the increased risk of revision due to dislocation for the PA compared with the DLA (Garellick et al. 2012). None of the surgical approaches were considered superior in adult patients undergoing THR for OA, which was consistent with the Cochrane review by Jolles and Bogoch (2006). However, the SHAR advocated the use of the DLA in patients with risk factors for dislocation (Garellick et al. 2012). These reports likely influenced surgeons’ awareness and provided evidence for improvements in the PA surgical technique.

### Strengths and limitations

The SHAR has a high completeness on primary THR ranging from 98% to 99% and intentionally includes all reoperations and not only revisions. The SHAR has nationwide coverage, which makes the results generalizable. Hence, geographical differences are not likely to affect the results. The inclusion criteria contribute to a more homogeneous study population. The choice of surgical approach for the selected population has most likely been influenced by the local tradition at each hospital rather than patient-specific attributes, which in turn affect the risk of reoperation due to dislocation.

The lack of data on BMI and ASA from 1999 to 2006 (given that the SHAR did not start the registration of those variables until 2008) means we were not able to adjust for these confounding factors. Another limitation pertains to the lack of information on the extent of soft tissue repair or the orientation of components. Furthermore, we did not include information on prosthesis type or surgeons’ experience. The SHAR does not capture information on closed reductions. It is unlikely, however, that dislocations can be treated non-surgically at higher success rates with one or the other approach. To what extent the recommendations from the SHAR’s Annual Reports may have caused a selection bias, with complex OA cases with higher anticipated dislocation risk having been operated through a DLA, is uncertain.

The skewed accumulation of bigger head sizes and more cemented THRs in the PA group may have favored this surgical approach regarding dislocation survival; however, the accumulation of fewer women and older patients may have disfavored it. The differences between the groups are statistically significant but seemingly small and adjusted for in the statistical analyses.

### Conclusion

In this nationwide observational study, we demonstrate that the historic increased risk of reoperation due to dislocation within 2 years for the PA compared with the DLA has declined substantially in contemporary Swedish THR practice. We believe enhanced surgical technique for the PA, increased awareness of the historically higher dislocation risk of PA, or possibly selection bias may explain this finding. The PA was associated with lower risk of reoperation due to all causes in 2007–2014 compared with the DLA. These findings warrant further research.

OR and MM conceived and designed the study. DO and SN performed statistical analysis. OS drafted the manuscript. All authors interpreted the results, contributed to the discussion, and reviewed the manuscript.

*Acta* thanks Stephan M Röhrl for help with peer review of this study.
